# “Tumor immunology meets oncology” (TIMO), 18 April–20 April 2024, in Brandenburg an der Havel, Germany

**DOI:** 10.1007/s00262-024-03877-1

**Published:** 2024-11-13

**Authors:** Barbara Seliger

**Affiliations:** 1 Brandenburg Medical School (Theodor Fontane), Institute of Translational Immunology, Gertrud-Piter-Platz 7, 14770 Brandenburg, Germany; 2 Institute for Medical Immunology, Martin Luther University of Halle-Wittenberg, Magdeburger Straße 02, 06112 Halle (Saale), Germany; 3 Fraunhofer Institute for Cell Therapy and Immunology (IZI), Leipzig, Germany; 4 Faculty of Health Sciences Brandenburg, Brandenburg Medical School Theodor Fontane, Brandenburg, Germany

**Keywords:** Immune escape, Immune response, Immunotherapy, TIMO 2024, Tumor microenvironment

## Abstract

The TIMO meeting XVIII 2024 covered both basic and translational tumor immunological topics, which were presented by national and international scientists and clinicians.

## Introduction

The yearly “Tumor Immunology Meets Oncology” (TIMO XVIII) meeting 2024 took this year for the first time place at the City Hall of Brandenburg an der Havel, Germany. National and international experts in basic tumor immunology and immune oncology presented novel data on immune escape mechanisms, (immuno)therapy resistances and the composition of the tumor microenvironment (TME) as well as a number of preclinical and clinical studies using different immunotherapies as monotherapy or combination therapy. In addition, next to talks about the tumor immune surveillance and escape processes resulting in acquired therapy resistances, possible options to monitor and revert the immune escape phenotype and resistances were given, in particular interventions by combining current immunotherapies together or with other treatment modalities. Based on the recent advances in artificial intelligence (AI), high-throughput technologies and their analysis with different bioinformatics tools, one session focused on AI approaches using different strategies.

The TIMO XVIII 2024 brought together researchers and clinicians from basic science to clinical applications, the latter focusing on immunotherapies using immune checkpoint inhibitors (ICPis) as well as cellular therapies. It became obvious that a prerequisite for understanding the biology of resistance mechanisms is an increased and in-depth knowledge of the molecular and immunological profile of tumors. The identification of novel biomarkers or resistance mechanisms will help to monitor, stratify and select patients, for individualized therapy, which then might lead to an increased treatment success. Next to the main symposium, TIMO offers also a comprehensive workshop, in particular for young scientists and clinicians, which also covered the topics of the main symposium. Furthermore, technological advances applied for immunomonitoring were presented by some companies.

After a warm welcome of the dean of the faculty as well as a representative of the city, the scientific program of TIMO XVIII 2024 started by the organizer Barbara Seliger (Brandenburg an der Havel, Germany), who focused her talk on extracellular matrix (ECM) proteins in cancer immune evasion and their possible reversion. Although ECM components, like the small and leucine-rich proteoglycans (SLRPs), play a role in the tumor development, their consequence on the immunogenicity and immune escape of tumors has not yet been well described. For example, the expression of the proline/arginine and leucine-rich protein (PRELP) is reduced in human tumors of distinct origin including melanoma compared to corresponding normal tissues. This was associated with an impaired human leukocyte antigen (HLA) class I expression accompanied by a reduced overall survival (OS) of patients. PRELP overexpression reverted the suppression of HLA class I surface expression and increased CCL5 secretion. Next to PRELP, the expression of the SLRP biglycan (BGN) was also downregulated in many tumor entities compared to healthy tissues as well as upon oncogenic transformation by HER2/neu. This was also confirmed by in silico analysis of HER2/neu^+^ breast cancer samples. Overexpression of BGN in HER2/neu transformants resulted in a reduced migration and cell proliferation as well as an induction of HLA class I surface expression accompanied by an increased expression of HLA class I antigen processing machinery (APM) components, which is in line with an increased immunogenicity and reduced tumorgenicity of BGN-overexpressing HER2/neu^+^ cells in vivo. RNA sequence (RNA-seq) analysis of BGN^high^ versus BGN^low^ HER2/neu-transformed cells identified a large number of differentially expressed genes, which were involved in various signal transduction cascades, including the mTOR/eIF-e4 pathway, which is suppressed by BGN overexpression. Despite its tumor suppressive effect in the myelodysplastic syndrome (MDS) and in secondary acute myeloid leukemia (sAML), BGN overexpression was accompanied with a worse prognosis. Thus, the ECM components are involved in both tumor progression and tumor suppression depending on the tumor (sub)type, which also shape the composition of the TME and influence patients’ survival. Therefore, the development of therapeutic strategies for the modulation of ECM components is urgently needed.

Vincenzo Bronte from the Istituto Oncologico Veneto in Padua, Italy, gave insights into the immune evasion in pancreatic ductal adenocarcinoma (PDAC), which is a clinical challenge due to late diagnosis, low resection chances and 80% relapse after surgery with low five years patients’ OS. This might be due to different factors, including the enforcement of an immunosuppressive tumor microenvironment. Understanding and targeting the immune deviation might thus offer promising therapeutic options. Among others, this includes reprogramming cancer-associated fibroblasts (CAFs), tumor-associated macrophages (TAMs) as well as myeloid-derived suppressor cells (MDSCs). Vincenzo Bronte focused on neutrophils and neutrophil extracellular traps (NETs), which are enriched in the TME of PDACs. Previous studies demonstrated that the knockout of arginase1 (ARG1) could enhance tumor rejection upon adoptive cell therapy (ACT). ARG1 is localized and activated in NETs, which are released by tumor-activated neutrophils in PDAC patients. Inhibition of ARG1 by specific monoclonal antibodies (mAbs) enhanced the efficiency of ICPi. NET neutralization could thus favor the function of infiltrating T lymphocytes in PDACs, turning a “cold” into a “hot” environment. In addition, he showed that Claudin 18 plays a role in shaping the immune contexture in PDAC and might serve as a prognostic marker and target, since it is expressed during the early stages of tumor progression and is associated with CD8^+^ T cell immune infiltration.

Another interesting talk was provided by Ahmed Al-Samadi from the University of Helsinki, Finland. His work is based on head and neck squamous cell carcinoma (HNSCC), a disease with an increased incidence worldwide. Despite an improved survival, there is an urgent need to enhance treatment efficacy, which is mainly surgery followed by radiation, chemotherapy or targeted therapies and recently also by immunotherapies. So far, the sequence and combinations of the therapeutic options have not yet been investigated in detail. Indoleamine 2,3-dioxygenase (IDO1) is overexpressed in HNSCC, which impacts the immune cell responses. Targeting of IDO1 in combination of ICPi has shown limited efficacy in melanoma but promising results in HNSCC. Furthermore, the IDO1 inhibitor can induce the infiltration of CD4^+^ T cells and NK cells. To unravel the molecular mechanism of the IDO inhibitor, HNSCC cells were cocultured with patients’ CD4^+^ T cells leading to a significantly increased secretion of MCP1 and GM-CSF and a decrease in Treg markers.

Dimitrios Mougiakakos from the Department for Hematology, Oncology and Cell Therapy of the University Hospital in Magdeburg, Germany, talked about the impact of DNA damage on antileukemic T cell activity. This is based on the fact that oxidated stress represents an effective immune escape mechanism of cancer and is enhanced during aging, which is accompanied by a reduced immune cell function. Concerning hematological diseases, allogeneic stem cell transplantation (SCT) is the most successful immunotherapy, but next to graft versus leukemia (GvL), there exist also graft versus host (GvH) effects. Furthermore, oxidative stress and DNA damage accumulate and trigger oxidative stress upon allogeneic (allo) SCT by increasing oxidated proteins, for example, lipids, oxidated DNA and the nuclear factor 2 (NRF2), exhibiting multiple regulatory roles including the depletion of defense against oxidative stress and oxidative stress after allo-SCT. OxDNA is reconstituted in T cells over time after allo-HSCT, which is associated with patient’s characteristics and correlated to systemic circulating OxDNA. The gene expression profile of reactive oxygen species (ROS)^high^ and ROS^low^ T cells revealed a distinct transcriptome profile, which was associated with cell proliferation and metabolism. Despite ROS^high^ T cells expressed higher levels of activation markers, they represent a more exhausted phenotype, which was accompanied by a reduced ability to eliminate acute myeloid leukemia (AML) cell lines as well as AML blasts. Increased DNA oxidation in T cells at day plus 60 after SCT were associated with disease relapse, but not with GvHD. This was accompanied by a shorter patients’ survival. Furthermore, prevention of DNA oxidation improved the proliferative capacity and functionality of T cells.

Stefan Glück from the Department of Hematology, Oncology, Immunology Miami Beach in Florida, USA, gave an excellent overview about the updated hallmarks of cancer first described 2000 and extended over the years and their role in immune oncology (IO). These include senescence, microbiome, non-mutational epigenetic reprogramming, unlocking phenotypic plasticity, tumor-promoting inflammation as well as avoidance of immune destruction. These hallmarks could be targeted by different strategies, such as inhibition of growth factor receptors, reactivating tumor suppressive pathways and enhancing cell death by overcoming apoptosis resistance, targeting telomerase or non-mutational epigenetic reprogramming as well as chronic inflammation. Concerning the cancer immune cycle one focus was the regulation of immune response by its induction, potentiation, adaptation and/or maintenance. Cancer involves complex changes in the TME, the tumor itself, priming of the metastatic niche and in the repertoire of circulating metabolites and cytokines. The development of high-throughput technologies including the assessment of the tumor mutational burden (TMB) by next-generation sequencing (NGS) has revolutionized the knowledge of the molecular and immunological features by in-depth characterization of different tumor (sub)types and also allowed the prediction of therapy responses. For example, the level of TMB associated with the response to immunotherapy. The 14 hallmarks of cancer, which are often associated with a worse patients’ survival, are characterized by a rapid and effective adaption to changes in cell stressors with the immune interface as important feature for immune dysfunction responsible for the initiation and progression of tumor cancer. An increased knowledge of the inflammation and immune cell composition is essential for designing new therapeutic strategies, which could overcome the strong immune dysfunction and increased neoplastic features of cancer.

Andreas Lundqvist from the Karolinska Institutet in Stockholm, Sweden, gave insights into the regulation of NK cell activity in solid tumors with specific focus on breast cancer and sarcoma and the CD73 immune checkpoint, which defines regulatory NK cells within the TME. CD73 expression is increased in tumor-infiltrating NK cells compared with peripheral blood NK cells, and these cells display as suppressive capacity of T cells. In addition, these tumor-associated NK cells are able to drive 4-BBL-positive melanoma cells toward epithelial–mesenchymal transition (EMT) and metastatic disease. Furthermore, Dr. Lundqvist showed that NK cells produce high levels of IL6 within MHC class I low tumors, leading to an induction of monocyte-derived MDSC, thereby inhibiting T cell responses. Depletion of NK cells producing IL6 limited the induction of MDSC, while targeting of the IL-6R restored T cell proliferation and reduced tumor dissemination. The NK cell-mediated induction of mo-MDCs influenced the immune responses to ICPi demonstrating an association of NK cells with ICPi responders and nonresponders.

Ebba Sohlberg from the Karolinska Institute in Stockholm, Sweden, discussed immunotherapy with allogeneic NK cells and specifically the power of NK cell therapy using a specialized adaptive NK cell subset. Many studies have described the diversity in the NK cell repertoire and a recent in-depth profiling of NK cells via a transcriptional reference mapping characterized the NK cell repertoire from CD56 bright to early CD56 dim, intermediate CD56 dim and late CD56 dim to adaptive NK cells present in CMV-seropositive individuals. The adaptive NK cell subset has many advantages that make them suited as a source for cellular therapy. Adaptive NK cells have enhanced antibody-dependent cellular cytotoxicity (ADCC) and express high levels of effector markers, but decreased expression of immune checkpoint (ICP) molecules. Most importantly, the finding that adaptive NK cells preferentially express a single-self KIR receptor opened an avenue of development in relation to alloreactivity and “missing-self” responses. So far, the missing self-theory of NK cell activation has not yet been fully tested in the clinic despite being known for over 40 years, as only a low fraction of conventional NK cells can exert these responses due to inhibition by their diverse KIR repertoires. In a therapy based on single-KIR adaptive NK cells, the HLA-C of the patient and the KIR of the adaptive NK cells could be mismatched to unleash “missing-self” responses of all transferred NK cells. A GMP-compatible protocol was developed to selectively expand adaptive NK cells using an HLA-E-positive K562 feeder cell line in an 11-day culture with IL-2. The generated “ADAPT-NK” cells were NKG2C^+^single-KIR^+^ and highly functional in an AML mouse model, and against primary AML cells in the HLA-C/KIR mismatched setting. Further, due to lack of inhibitory NKG2A expression in favor of activating NKG2C, ADAPT-NK cells were triggered by HLA-E overexpressing targets. ADAPT-NK cells also showed potent ADCC in combination with various immune engagers and tumor targets, including solid tumor patient-derived cell lines. These results led to translation of adaptive NK cell therapy into the clinics. For this, “super donors” with large fractions of adaptive NK cells were recruited and cell material was banked. ADAPT-NK cell manufacturing was established, and plans are in place for a phase I trial for HLA-C homozygous patients with refractory AML/MDS. The patients will receive the manufactured adaptive NK cells by infusion, and the NK cells will be supported in vivo by IL-2. Immunomonitoring will take place for up to 90 days carefully profiling the infused NK cells, the recipient immune system and the tumor compartment. Work is ongoing to further improve the adaptive NK cell therapy by introducing specificity with immune engagers or CAR engineering.

Ofer Mandelboim from the Haddassah University, Jerusalem, Israel, presented novel data from the bench to the clinic regarding treatment of tumor patients with an anti-poliovirus receptor (PVR) antibody (Ab). This novel approach is important, since the available immunotherapies are still limited with 20 to 30% overall response rates of patients, which is related to nonredundant pathways leading to escape mechanisms. Therefore, therapies targeting these alternate pathways are required. Nectin4 has been shown as an important escape pathway upon programmed death ligand 1 (PD-L1) therapy and is thus a potential target in immune oncology. The PVR, named also CD155, a member of the nectin-like protein family, is involved in different physiological cellular processes like cell differentiation, migration, proliferation and immune responses. Its immune regulatory potential is due to the interaction with the co-stimulatory receptor DNAM-1, the inhibitory receptor TIGIT and CD96. PVR is overexpressed in many human malignancies, but lack expression in normal healthy tissues. A high-affinity Ab binding to PVR was developed, which blocks the interaction with its inhibitory receptors TIGIT and CD96, thereby stimulating CD8^+^ and NK cell effector function and inhibiting tumor growth in vitro. Based on these results, a first clinical phase I trial using the anti-PVR Ab was set up and the first patients enrolled. These data will provide information whether PVR is a suitable target for therapy.

Lorenzo Galluzzi from the Weill Cornell Medical College in New York, USA, focused his talk on immunogenic cell death (ICD) as driven by radiotherapy. ICD requires different steps and can be induced by pathogens, as well as immunogenic chemotherapeutics, radiation therapy (RT) as well as by necroptosis inducers. The ICD-related secretion of damage-associated molecular patterns and cytokines promotes antigen presentation by dendritic cells (DCs) in the context of optimal immunostimulation, which can result in systemic disease control upon local therapy, as in the case of so-called abscopal responses to RT. Importantly, mitochondrial apoptosis is known to shown to contribute to RT cytotoxicity. Moreover, mitochondrial outer membrane permeabilization as driven by RT supports ICD by enabling mitochondrial DNA accumulation in the cytosol and consequent type I interferon (IFN) secretion upon cGAS-STING signaling. This process, however, is inhibited by autophagy as well as by apoptotic caspases, both of which curb abscopal responses to RT in preclinical tumor models.

Mitchell Levesque from the University Hospital, Department of Dermatology in Zürich, Switzerland, showed any impressive multidimensional melanoma analyses, which were correlated to the clinical setting. The molecular features of the tumor help to identify, which patients respond to the targeted therapy, while the follow-up treatments are based on in particular molecular features of relapsing cells. There exists a significant transcriptional heterogeneity in melanoma and a phenotype switch from the melanocytic to the mesenchymal phenotype, characterized by distinct gene expression patterns. Over the recent years, different tools have been developed, which are important for biobanking followed by the application of various high-end technologies and data analyses. These include the fresh sample processing, also allowing ex vivo culturing of cells. First bulk molecular analyses are performed, followed single-cell analysis via flow cytometry, mass cytometry as well as single-cell RNA-seq. Dr. Levesque’s group has in his toolbox more than 500 patients, 1500 cores and validated melanoma cultures. The samples were obtained from primary melanoma, relapse or metastatic melanoma, before and after BRAF1/MEK inhibitor treatment as well as metastatic melanoma with a distinct immune cell infiltration pattern. He established an atlas of molecular features of melanoma cells for discovery and validation, which improved the precision medicine of this disease with focus on single-cell and functional analyses of melanoma. This approach allowed to distinguish between MEK inhibitor-sensitive and resistant phenotypes and also allow to target tumor heterogeneity. The MEK inhibitor-sensitive phenotype is dependent on the MAPK pathway, an intact metabolism and high ROS scavenging. In contrast, the MEK inhibitor-resistant phenotype is MAPK pathway independent, metabolically silent and has low ROS scavenging. × 3.

Marta Ligero Hernandez from the Else Kroener Fresenius Center of Digital Health in Dresden, Germany, discussed the potential of AI analyze and integrate routine clinical data including pathology, radiology and patients’ health records to predict the response to immunotherapy and enhance treatment selection. The deep learning approach is required for the assessment of the ICP status directly from raw immunohistochemical maps of stained slides, since the manual and computer-based readouts are not that reproducible. In addition, the performance of the model to predict ICP response was tested.

Wim Vos from radiomics.bio in Liège, Belgium, emphasized the importance of intra-patient and inter-tumor variability in the development of IO therapies. Advanced radiologic image analysis also known as radiomics offers new opportunities to address these challenges. It is well established that (i) approximately two-thirds of patients present with metastatic tumors at diagnosis, (ii) many cancers undergo mutations as they spread throughout the body and (iii) patients in early-phase drug development trials are often highly metastatic. The occurrence of heterogeneous response or dissociated progression, where different lesions react differently to treatment, ranging from disappearance to hyperprogression. Current clinical trials heavily rely on the response evaluation criteria in solid tumors (RECIST) or its adaptations to assess therapy efficacy. However, RECIST typically evaluates only a limited subset of lesions—five lesions at most, with no more than two per organ—which limit its sensitivity to therapy effects. Heterogeneous response is one situation where RECIST limitations become more flagrant. In contrast, radiomics allows for the segmentation and quantification of each individual lesion in standard radiological images (CT, MRI, PET), providing a more detailed analysis across four key domains: (1) size (e.g., volume), (2) shape (e.g., sphericity), (3) intensity (e.g., average Hounsfield units) and (4) texture (e.g., intra-tumor HU contrast). These quantifications, called radiomic features, can be tracked over time under specific treatment conditions, with changes in these features potentially serving as more comprehensive markers for drug efficacy. This individualized lesion analysis represents an effective method for "upsampling" in small clinical trials, as each patient may present with multiple lesions and is in particular valuable in early-phase clinical trials, such as dose-finding studies, addressing concerns raised by the Federal Drug Administration (FDA)’s Project Optimus initiative regarding overexposure to therapeutics beyond the point of maximal efficacy. Furthermore, radiomics-based statistical models enable the creation of predictive models for tumor biology and therapy response. These models have the potential to explain observed trial results and refine the selection of target patient populations. Despite its promise, radiomics is still an emerging field. Careful interpretation of published results is necessary, but further research is required to better understand the physical significance of radiomic features, their variability and the development of prediction models that are generalizable across different datasets.

Luis Zapata Ortiz from the Institute of Cancer Research, Center for Evolution and Cancer, London, Great Britain focus his talk on public available datasets of primary tumors and immune checkpoint-treated metastases and the development of an evolutionary metric, which will allow to stratify patients based on immune selection by analysis of the ratio of non-synonymous to synonymous mutations presented as immunopeptidomes by HLA molecules. This approach also allows to determine and validate the link between immune selection and T cell infiltration in primary immune-edited tumors and to distinguish between immune-edited versus immune escaped tumors enabling the prediction of response to immunotherapies, such as ICPi.

The keynote of this year’s TIMO was presented by Sumit K. Subudhi from the University of Texas MD Anderson Cancer Center in Houston, USA, summarizing different IO strategies to overcome the immunosuppressive TME using prostate cancer as a model. During the last years, a number phase III immunotherapy trials were implemented for the treatment of prostate cancer, which included vaccines as well as different ICPi alone or in combination with other options. Since the efficacy of these different strategies regarding a durable effect was limited, the clinical response to ICPi, such as anti-CTLA4, should be improved. Anti-CTLA4 therapy-induced T cell infiltration and Th1 responses in prostatic soft tissues, but also promoted upregulation of the inhibitory ICPs, PD1, PD-L1 and VISTA within the TME. Combination ICP blockade with anti-CTLA4 plus anti-PD1/PD-L1 treatment improved clinical responses in patients with measurable diseases, but a balance between efficacy and toxicity will be required to move this therapeutic strategy forward. However, compared to ICP monotherapy with anti-CTLA4 or anti-PD1 the combination failed to improve clinical outcomes in patients with bone predominant metastases. This may be attributed to the combination of ICP blockade increasing myeloid cells in the prostate bone marrow (BM) TME. The prostate BM TME exhibited a fibrotic phenotype characterized by a transforming growth factor (TGF) β pathway transcriptional signature and targeting elevated TGF-β1 levels in preclinical mouse models improved the efficacy of anti-CTLA4 therapy in a bone TME model. Interestingly, VISTA, but not PD-L1 was upregulated in the prostate BM TME after treatment with ICPi, which might explain the poor responses to a combined anti-CTLA4 plus anti-PD1/PD-L1 treatment in patients with bone predominant metastases. In exosomes, an increased transcription of myeloid pathway genes was found, including those of the adenosine pathway. Targeting of the adenosine pathway, known to be linked to VISTA and TGF-β signaling that converges on myeloid cells, increased clinical responses. These data improved understanding of the immunosuppressive TME associated with resistance mechanisms within the BM in prostate cancer, which were mediated by IL6 and TGF-β as well as by myeloid cells. Bispecific T cell engagers could overcome the immunosuppressive TME of prostate cancer as demonstrated by antitumor responses associated with upregulation of total T cell transcriptional signatures of in particular the MHC class I antigen presentation pathway, the T cell receptor (TCR) as well as the interferon (IFN)-γ signaling in lesions of responders versus nonresponders. However, limitations of bispecific CD3 T cell engagers include the cytokine release syndrome, T cell exhaustion, activation-induced cell death and the lack of memory responses. In-depth analyses of responses to bispecific CD28 T cell engager plus anti-PD1 Abs demonstrated distinct responses associated with the function of new clonal T cell subpopulations within the metastatic prostatic TME. However, the distinct mechanisms between CD3 versus CD28 bispecific T cell engagers ati-CD3 and anti-CD28 T cell-specific Abs have to be dissected, which induce T cell infiltration into the TME. This strategy will likely have to be combined with therapeutic agents that overcome adaptive resistance mechanisms by targeting myeloid-associated pathways, such as TGF-β, VISTA and/or adenosine.

Joanne Lysaght from the Cancer Immunology Immunotherapy Group of the Trinity College in Dublin, Ireland, described the complexity behind the response rate to ICPi. Since only a limited number of patients do respond to this treatment, the impact of the TME and standard of care regimens on T cells and their potential influence on the response to ICPi has to be analyzed. Parameters influencing the patients’ response to ICPi include immune cell infiltration, IFN-γ signaling, epigenetic modification, TMB, HLA class I expression, gut microbiota, ICP molecule expression as well as acidosis, hypoxia and nutrient deprivation. Furthermore, the TME-induced stress results in significant phenotypic and functional changes in T cells, which may then interfere also with the response to ICPi. Nutrient deprivation and hypoxia both affect pro- and anti-inflammatory cytokine expression, while an increase in acidity enhances the expression of ICPi molecules. Furthermore, there exist sex differences in immunity, which might also affect the efficacy of anticancer immunotherapy. Using esophageal carcinoma (OAC) as a model, the current treatments of this disease were analyzed for changes in ICP expression by comparing perioperative chemotherapy with neoadjuvant radiotherapy. Both therapies promote a pro-inflammatory T cell profile, while creating a therapeutic vulnerability via upregulation of ICP. A combination of RT with ICPi results in a significant increase of tumor cell killing. Interestingly, OAC chemotherapy regimen upregulates ICP on the surface of cells, thereby enhancing a more immune resistant phenotype, which is maintained over time, while anti-ICP enhanced the cell death and chemotherapeutic toxicity in OAC cells. Thus, there is an urgent need to identify the best timing to incorporate ICPi for OAC treatment as well as in identifying the best combination treatment.

Christian Münz changed the topic and talked about the immune control of oncogenic human γ herpes viruses with focus on Epstein–Barr virus (EBV) and Kaposi sarcoma virus (KHSV). EBV-associated diseases arise either from B cell infection or from atypical target cell infection. Latent and lytic EBV infection programs of associated tumors are present in healthy virus carriers. The primary immune deficiencies identified hallmarks of EBV-specific immune control. Furthermore, EBV synergises with other infectious diseases for its lymphomagenesis. KSHV infection drives plasma cell differentiation in EBV-infected humanized mice and preferentially persists with EBV coinfection thereby increasing the EBV-associated lymphomagenesis. KSHV persists in EBV-infected cells of the large B cell lineage and its coinfection drives lymphoma. Furthermore, a crosstalk between KSHV-infected B cells and T cells exists in the spleen of humanized mice. Coinfection drives effector and central memory CD8^+^ T cell expansion as well as IgM responses in humanized mice. In KSHV and EBV co-infected mice that cannot activate the lytic EBV infection, T cell responses preferentially recognizing double-infected B cells can be detected. Furthermore, T cells from such mice recognize KHSV open reading frames, with K6 as an antigen. T cell depletion increases both EBV and KSHV infection in vivo*,* while KSHV-specific CD8^+^ T cell clones can kill double-infected B cells in vitro and in vivo.

Thomas F. Gajewski from the University of Chicago, USA, presented his work on myeloid cells in the TME and their effect on T cell-based immunotherapy efficacy. The T cell-inflamed and non-inflamed TME represent two categories of immune escape. An increased activity of Abs directed against programmed death receptor 1 (PD1) was associated with a T cell-inflamed TME at baseline, which is generated by the host STING pathway type I IFN and DC. Furthermore, tumor cell intrinsic β-catenin activation prevents host antitumor response by a failure to recruit Batf3 DCs. The Batf3 lineage DC are required during PD1/PD-L1 inhibition to provide positive signals for TILs reinvigoration within the TME and anti-PD-L1 efficacy is lost upon selective depletion of DCs within the TME immediately prior to radiation. Spatial analysis of CD8^+^ T cells and Batf3 DC revealed a nonrandom distribution associated with an anti-PD1 efficacy, but a CD8^+^ T cell and DC proximity and the associated with mutually relevant chemokines and activation states as well as the interaction of Batf3 DC and 4-1BB with CD8^+^ T cells are required for anti-PD1 efficacy. In order to revert cold, nonimmune cell-infiltrated tumors, a STING agonist in the presence of pembrolizimab only has a limited clinical efficacy and fails to control tumors, while in vitro activated intra-tumorally injected DCs revert resistance to immunotherapy. Furthermore, the combination of intra-tumoral FLT3L and DMXAA improves tumor control in mice treated with anti-PD-L1 plus anti-CTLA4 Ab. An inter-patient heterogeneity exists, which regulates the T cell-inflamed TME, in particular the suppressive myeloid cells. Since β-catenin-expressing tumors expand M2-like macrophages and T cells near M2 macrophages lack the activation signature of T cells near DC, β-catenin induces an unfavorable suppressive TME. Furthermore, unfavorable gut microbiota in mice exhibit a shift from M1 to M2 macrophages and MDSC granulocytes, while a germ line variant in phosphokinase C (PKC) δ augmented antitumor immunity driven by the M2 to M1 transition. This is underlined by a shift from the M2 to M1 macrophage phenotype in PKC δ knockout mice and improves tumor control in vivo by conditional depletion of PKC δ in macrophages. Thus, anti-PD1 efficacy is favored by an inflamed TME and depends on the interactions between Batf3^+^ DC and CD8^+^ T cells within the tumor sites. The T cell-inflamed TME is regulated by tumor cell intrinsic oncogenic events as well as the composition of the commensal microbiota and germ line polymorphisms in immune regulatory genes. These features functionally influence important myeloid cells in the TME. Strategies to promote Batf3 DC recruitment and activation as well as interventions to shift the M2 macrophage phenotype to M1 have therapeutical potential.

Maria Goulielmaki from the Cancer Research Center at Saint Savas Cancer Hospital in Athens, Greece, identified predictive biomarkers in non-small cell lung cancer (NSCLC) after treatment with anti-PD-L1 Ab by analyzing the dynamics of the TCR repertoire. Since a number of predictive biomarkers of immunotherapy responses have been suggested the immunotherapy-induced alteration in the TCR V β repertoire and its potential as prognostic marker was analyzed in sequential peripheral blood samples of patients with unresectable, PD-L1 positive stage III NSCLC treated with anti-PD-L1 mAb post-radical radio- and chemotherapy, while a control group received alternative treatments. Analysis of the TCR repertoire in blood and tumor tissues of 36 NSCLC patients at baseline demonstrated shared clonotypes, which highly varied in their number, from 4 to 378 with large differences in the V gene usage. Thus, significant alterations regarding the TCR repertoire dynamics were identified between the patient's tumor and blood samples at baseline with a reduced number and diversity in the CDR regions of tumor versus blood. Differences in the V gene usage were found in the blood pre- versus post-immunotherapy with an increase in the frequency of new TCR clonotypes post immunotherapy. However, no link between TCR clonality and survival, but survivors had increased frequencies of certain V genes at baseline and loss of clonotypes compared to non-survivors. The preexisting immunity did not correlate with the TCR repertoire dynamics, but patients with preexisting immunity and lost clonotypes had significantly improved progression-free survival (PFS). In contrast, significant differences in the TCR composition were identified based on the TMB with an improved PFS of patients with high TMB and lost clonotypes. However, a follow-up of this study, a validation cohort to confirm these data as well as the functional characterization of the identified TCR clonotypes are urgently needed.

Markus Maeurer from the Champaulimaud Foundation in Lissabon, Portugal, talked about the antitumor directed T cell responses in epithelial cells by determining the inter-patient as well as intra-tumoral variation, which could be associated with alterations in bacteria, fungi and viruses. In order to assess the intra-tumoral heterogeneity, a complex workflow was established by using tissue material from different well-defined areas of the tumor, which were either directly formalin-fixed paraffin-embedded (FFPE), fresh frozen or directly natively used to isolate tumor-infiltrating lymphocytes (TILs). Different regions of the tumor tissue were dissected and then analyzed for T cell infiltration and tumor nests, followed by immunohistochemical analysis using CD3 and IFN-γ as markers. The TILs were then expanded and monitored for their phenotype, efficacy in mounting antitumoral immune responses and cytokine release. Since the TIL culture expansion will allow to infuse high number of cells into patients, a sophisticated workflow was developed. Reliable expansion of antigen-reactive TILs from epithelial cancer as well as TCR γδ T cells was demonstrated. However, there exist a different quality of antitumoral immune responses.

Francesco Marincola from Sonata Therapeutics in Boston, USA, suggested a shift in the cancer treatment paradigm by introducing cancer-specific medicines that modulate the cancer multicellular network. The current treatment paradigm is generally bimodal: either immunotherapies, which often fail due to lack of cancer specificity, leading to toxicity; or cancer-targeted approaches, whose precise specificity creates an avenue for cancer escape. To overcome these limitations, Sonata is developing Network Medicines. Network Medicines are specific for cancer cells, both killing them and inducing the release of key cytokines and chemokines that drive an amplified immune mechanism, inhibiting the chance of escape. Successful immune-mediated cancer therapy has four requirements to induce cancer rejection: (i) presence of immune cells and their trafficking into desert tumors, (ii) penetration of immune cells into immune excluded tumors, (iii) persistence of activity mediated by fitness and stemness and (iv) preponderance over immune suppression. There is an urgent need for therapeutics that simultaneously address all four “Ps” required for cancer rejection. Direct and indirect cancer killing mechanisms are both necessary to achieve this: thus, the cancer cell itself needs to release signals that coordinate the multicellular network, thereby exponentially increasing tumor cell killing. Based on this assumption, reprogramming of cancer cells to become the coordinators of the cure is postulated. Different therapeutic modalities are available to achieve this goal, with genetic payloads as one option. SNT-3012, a unique mRNA-based therapeutic, drives complete tumor regression in models of hot and cold tumors, inducing T cell activation, proliferation and memory. Virus-like particles may be able to selectively and specifically deliver mRNAs into cancer cells. In order to address immune escape, smart chimeric antigen receptor (CAR)-T cell therapies should also be generated, which overcome the current challenges. Aside from delivery of cells or genetic material, Sonata has demonstrated that cancer cell reprogramming may also be achieved by inhibition of single cancer-intrinsic targets, creating opportunity for small molecule therapeutics. Currently, Sonata has identified several novel Network Medicine small molecule targets to treat NSCLC, with PDAC as a next potential area for exploration. In sum, Network Medicines meet all important criteria for the development of safe, effective and durable cancer therapies.

Maria Fortunata Lofiego from the Center for Immuno-Oncology of the University of Siena, Italy, talked about the immunomodulatory potential of epigenetic drugs in melanoma. This is an important topic, since some tumors develop primary resistance mechanisms to immunotherapies and exhibit only a low frequency of response, while others develop novel escape mechanisms leading to secondary resistances, which has been documented across a variety of tumor types and has been extended to the response of the majority of cancer patients. Non-mutational epigenetic reprogramming which is maintained by the DNACytokine-5-methyltransferase 1 (DNMF1) is an important hallmark of cancer, but unlike genetic mutations, epigenetic modifications are reversible and exhibit high plasticity. Among different classes of epigenetic drugs, DNMT inhibitors (DNMTi) are particularly effective at increasing the immunogenicity of tumor cells by inducing or upregulating tumor-associated antigen expression, HLA class I, APM and IFN pathway components, and co-stimulatory molecules, leading to enhanced cytotoxic T lymphocyte (CTL)-mediated tumor recognition. To explore whether the immune remodeling of neoplastic cells by epigenetic drugs can be used to design novel immunotherapeutic approaches, different preclinical mouse models were exploited to investigate the immunomodulatory activity of DNMTi in combination with immunomodulating mAbs. The promising findings from these models prompted the investigation of the clinical relevance of this novel combination. Consequently, the first worldwide epigenetic-base immunotherapy trial, the NIBIT-M4 trial (NCT02608437), was designed and conducted. This phase Ib dose-escalation trial enrolled patients with advanced melanoma receiving guadecitabine in combination with the anti-CTLA-4 antibody ipilimumab. Initial results showed that the combined treatment led to a global reduction in tumor DNA methylation, resulting in the upregulation of double-stranded RNA, particularly derived from transposable elements (TEs). Upon translation into proteins, these TE can be presented as peptides via the MHC class I surface antigens. Integrated analysis of promoter methylation and gene expression data from responders (R) versus nonresponder (NR) patients at different timepoints, demonstrated a more immunogenic tumors in R, which showed progressive enrichment from baseline to week 12 in biological processes related to immune pathways in R, compared to processes related to skin development in tumors from NR patients. In light of these findings, glioblastoma multiforme (GBM), given its association with low T cell-mediated immunity, increased proliferation and invasion, and defective expression of MHC class I antigens, was used as a model to investigate the immunomodulatory potential of DNMTi. The epigenetic drug treatment enhanced the expression of several genes involved in immune cell recognition, activation, and proliferation of NK and NK T cells, while downregulating cell proliferation, metabolic processes and the hippo signaling pathway. In summary, DNMTi can modulate the GBM phenotype to a more immunogenic state, potentially improving responsiveness to immunotherapy by activating transcriptional factors involved in immune recognition, as well as innate and adaptive immune responses. Overall, cancer epigenetic remodeling represents a promising strategy to increase the efficacy of immunotherapies and supports the rationale for developing new DNMTi-based combinational immunotherapeutic approaches for the treatment of different cancer histotypes.

Bernard A. Fox from the Earle A. Chiles Research Institute and Providence Cancer Institute in Portland, USA, talked about a novel class of cancer antigens presented by MHC class I, the so-called “dark matter” as targets. Termed as “alternative cancer neo-antigens” some are only expressed by cancer cells, but not in corresponding normal tissues and present noncanonical non-mutated peptidomes. Some dark matter appears to be shared by different cancers and a number are associated with a worse patients’ outcome. Although these antigens have only recently been discovered, since they are not recognized by endogenous immunity and require in vitro priming or vaccination, they have a significant potential for novel TCR-based and next-generation cancer vaccines. Their immunogenicity has been explored demonstrating that a TCR against one of these peptide antigens was able to recognize melanoma, ovarian as well as gastric cancer. Bernard Fox’s group generated a novel vaccine named Dribble (DPV-001) vaccine containing both canonical (> 100 shared antigens) as well as short-lived proteins and some noncanonical dark matter, which was used in a clinical trial in NSCLC leading to increased T cell and B cell responses targeting relevant cancer antigens. Vaccination induces immunity to a wide range of shared cancer antigens and its combination with anti-PD1 and anti-GITR targeting CLEC9 increases the number of TILs. Furthermore, post-vaccine TILs expressed high levels of IFN-γ, granzyme B, CD39/CD103 as well as of LAG-3 and TIM-3.

Zlatko Trajanoski from the Medical University of Innsbruck, Austria, talked about dissecting the tumor microenvironment at single-cell level. The advent of single-cell RNA-seq technologies opened fundamental new avenues to dissect cancer heterogeneity. His laboratory recently developed a novel computational approach to dissect the cellular diversity in the TME at high resolution by integrating single-cell expression profiles from multiple studies using cutting-edge machine learning and AI methods, and created a high-resolution single-cell transcriptome NSCLC atlas. This atlas offers superior coverage of histological and clinical variables and provides a rich resource for dissecting cellular diversity in the TME. Then bulk RNA-seq data from The Cancer Genome Atlas was used and a novel computational method to evaluate the association of single-cell transcriptomic signatures with survival data and response to therapy. The exploitation of this high-resolution atlas provided novel biological insights that are the basis to improve therapy in NSCLC patients.

In sum, the TIMO meeting provided a unique opportunity of researchers and clinicians to share novel insights into tumor-intrinsic and extrinsic immune escape mechanisms, distinct technologies to assess the TME and into the improvement of immunotherapies. In addition, bioinformatics tools for the identification of biomarkers and innovative clinical trial designs were presented, which accelerate the clinical translation.

## Data Availability

No datasets were generated or analyzed during the current study.

## Abbreviations

Ab: Antibody
ACT: Adoptive cell therapy
ADCC: Antibody-dependent cellular cytotoxicity
AI: Artificial intelligence
AML: Acute myeloid leukemia
APM: Antigen processing machinery
ARG1: Arginase1
BGN: Biglycan
BM: Bone marrow
CAF: Cancer-associated fibroblast
CAR: Chimeric antigen receptor
CTL: Cytotoxic T lymphocyte
DC: Dendritic cell
DNMF1: DNA cystosine-5-methyltransferase 1
EBV: Epstein–Barr virus
ECM: Extracellular matrix
EMT: Epithelial–mesenchymal transition
FDA: Food and Drug Administration
FFPE: Formalin-fixed paraffin-embedded
GBM: Glioblastoma multiforme
GvH: Graft versus host
GvHD: Graft versus host disease
GvL: Graft versus leukemia
HLA: Human leukocyte antigen
HNSCC: Head and neck squamous cell carcinoma
ICD: Immunogenic cell death
ICP: Immune checkpoint
ICPi: Immune checkpoint inhibitor
IDO1: Indoleamine 2,3-dioxygenase
IFN: Interferon
IO: Immune oncology
KHSV: Kaposi sarcoma virus
mAb: Monoclonal antibody
MDS: Myelodysplastic syndrome
MDSC: Myeloid-derived suppressor cell
NET: Neutrophil extracellular trap
NGS: Next-generation sequencing
NRF2: Nuclear factor 2
NSCLC: Non-small cell lung cancer
OAC: Esophageal carcinoma
OS: Overall survival
PBMC: Peripheral blood mononuclear cell
PDAC: Pancreatic ductal adenocarcinoma
PD1: Programmed death receptor 1
PD-L1: Programmed death ligand 1
PFS: Progression-free survival
PKC: Phosphokinase C
PRELP: Proline/arginine and leucine-rich protein
PVR: Poliovirus receptor
RECIST: Response evaluation criteria in solid tumors
RNA-seq: RNA sequencing
ROS: Reactive oxygen species
RT: Radiation therapy
sAML: Secondary acute myeloid leukemia
SCT: Stem cell transplantation
SLRP: Small and leucine-rich proteoglycans
TAM: Tumor-associated macrophage
TCR: T cell receptor
TE: Transposable element
TGF: Transforming growth factor
TIL: Tumor-infiltrating lymphocyte
TMB: Tumor mutational burden
TME: Tumor microenvironment
Treg: Regulatory T cell
VLP: Virus-like particles

